# Utility of body mass index, waist circumference and waist-to-height ratio as screening tools for hyperglycemia in young people

**DOI:** 10.1590/2359-3997000000226

**Published:** 2016-11-07

**Authors:** Teresa Maria Bianchini de Quadros, Alex Pinheiro Gordia, Jorge Mota, Luciana Rodrigues Silva

**Affiliations:** 1 Universidade Federal do Recôncavo da Bahia Amargosa BA Brasil Curso de Educação Física da Universidade Federal do Recôncavo da Bahia, Amargosa, BA, Brasil; 2 Faculdade de Ciências do Desporto Universidade do Porto Porto Portugal Centro de Investigação em Atividade Física, Saúde e Lazer, da Faculdade de Ciências do Desporto da Universidade do Porto, Porto, Portugal; 3 Faculdade de Medicina Universidade Federal da Bahia Salvador BA Brasil Faculdade de Medicina, Programa de Pós-Graduação em Medicina e Saúde, Universidade Federal da Bahia, Salvador, BA, Brasil

**Keywords:** Pediatric obesity, anthropometry, pediatrics, hyperglycemia, diabetes mellitus

## Abstract

**Objectives:**

To evaluate the ability of BMI, WC and WHtR to discriminate hyperglycemia in young people, and to determine whether there is an increase in the accuracy with the addition of WC and/or WHtR to BMI.

**Subjects and methods:**

A cross-sectional study was conducted on 1,139 schoolchildren aged 6 to 17 years from Northeastern Brazil. Body weight, height, WC and fasting glucose levels were measured, and the BMI and WHtR were calculated. The presence of hyperglycemia was defined as a fasting glucose level ≥ 100 mg/dL.

**Results:**

The prevalence of hyperglycemia was 6.6%. Strong correlations were observed between the anthropometric indicators studied (BMI vs. WC = 0.87; BMI vs. WHtR = 0.87; WC vs. WHtR = 0.90). Hyperglycemia was more likely to be present in young people with excess weight (PR = 1.70), high WC (PR = 1.85), and high WHtR (PR = 1.91). The accuracies to discriminate hyperglycemia were significant, but low, for the individual (BMI = 0.56; WC = 0.53; WHtR = 0.55) and combined indicators (BMI + WC = 0.55; BMI + WHtR = 0.55).

**Conclusion:**

Our findings do not support the use of BMI, WC or WHtR as screening tools for hyperglycemia in children and adolescents.

## INTRODUCTION

The worldwide prevalence of overweight and obesity among children and adolescents has increased by about 47% in the last three decades (1). In parallel, a substantial increase has been observed in the prevalence of type 2 diabetes among young people (2). A growing body of evidence indicates that obese children and adolescents are more likely to present different cardiometabolic disorders (3), and that the maintenance of obesity from childhood to adulthood significantly increases the risk of type 2 diabetes (4).

Overweight and obesity are commonly evaluated in children and adolescents using the body mass index (BMI) because of its easy measurement and low cost. Additionally, reference values are available for comparison and the parameter permits continuous evaluation in adults (5). However, the relationship of this parameter with cardiometabolic alterations in young people can be questioned since the BMI estimates total body weight and not the quantity and distribution of fat. On the other hand, waist circumference (WC) and the waist-to-height ratio (WHtR) have gained ground in the evaluation of cardiometabolic risk factors in young people because these indicators propose to estimate central body fat (6,7). A systematic review including 61 studies found that the deposition of central body fat in children and adolescents increases the cardiometabolic risk, irrespective of the definition of abdominal obesity and anthropometric method adopted (3).

Although anthropometric indicators are attractive tools for the evaluation of cardiometabolic risk, it is not clear whether BMI, WC and WHtR are able to discriminate hyperglycemia in children and adolescents, or whether WC and/or WHtR confer additional utility to BMI. Therefore, the objective of this study was two-fold: 1) to evaluate the ability of BMI, WC and WHtR to discriminate hyperglycemia in young people, and 2) to determine whether the inclusion of WC and/or WHtR increases the accuracy of BMI to discriminate hyperglycemia in young people.

## SUBJECTS AND METHODS

### Population and sample

The present study is part of a school-based epidemiological study conducted in a city of Northeastern Brazil. The estimated population in 2012 was 34,845 inhabitants, with a human development index of 0.625. The population consisted of school-age children and adolescents of both sexes ranging in age from 6 to 17 years (6.0 to 17.99 years). The students were enrolled in the 1^st^ to 9^th^ grades of elementary school and in the 1^st^ to 3^rd^ year of high school in public and private schools of the city. According to data from the Municipal Education Department, 7,708 students were enrolled in 42 schools in 2011, including 40 public schools [13 urban (N = 5,207) and 27 rural (N = 1,853)] and two private schools (N = 648). The municipality studied comprises an area of 435,932 km^2^. For this reason, the Municipal Education Department divided the area of the municipality into six educational nuclei (one urban and five rural nuclei). Among the rural nuclei, the smallest possessed two schools and the largest seven schools in 2011. In the urban area, all schools were located in the same nucleus.

The representative sample size of the larger study was calculated using an estimated prevalence of 50% (for different outcomes), a 95% confidence interval, and a precision of 3 percent points according to Luiz and Magnanini (8). The estimated sample size was 971 children and adolescent; 20% of this number (n = 194) was added to account for possible incomplete data of the subjects or refusal to participate in the data collection.

The sample was selected in two stages, in which the “school” was the primary sampling unit and the “student” the secondary sampling unit. In the first stage, cluster sampling of the schools was used, with proportional stratification by type of school (urban public, rural public, and private) and by educational nucleus for schools in the rural area (in order to guarantee the geographic distribution of the sample in the rural area). Five urban public schools, five rural public schools (one per nucleus studied), and one private school were selected by drawing lots. The estimated sample size per extract was proportional to that observed in the study population (urban public: n = 787; rural public: n = 280; private: n = 98). In the second stage, the students were selected by simple drawing lots considering the number of individuals necessary per school to compose the sample, so that the number would be proportional to the number of students enrolled in each school. The study protocol was approved by the Ethics Committee of Faculdade Maria Milza (Permit No. 126/2011). Only students who voluntarily accepted to participate and whose parents or legal guardian had signed the free informed consent form were included in the study.

### Instruments and procedures

The data were collected between August 2011 and May 2012. The sociodemographic variables were obtained by self-report and included age, sex, and socioeconomic class. The last was estimated using the Brazilian Criterion of Economic Classification (9).

Body weight was measured with a Plenna digital scale (capacity of 150 kg) to the nearest 100 g. Height was measured with a Seca portable stadiometer (0 to 220 cm) fixed to the wall, to the nearest 0.1 cm. The two variables were measured using standard techniques (10) and were used to calculate the BMI. Overweight and obesity were defined using the cut-off values proposed by Cole and cols. (11). WC was measured with a non-elastic measurement tape to the nearest 0.1 cm according to procedures recommended by the World Health Organization (12), and was classified as normal or elevated (13). Height and WC were used to calculate the WHtR. A WHtR ≥ 0.5 was classified as high (14). Anthropometric assessment was performed in the morning by two examiners of the same sex as the students to avoid any embarrassment. The two examiners presented intra- and interobserver errors of less than 1% and 1.5% for all measures, which are acceptable according to the literature (15).

Venous blood samples (10 mL) for the measurement of blood glucose levels were collected at the schools in the morning after a 12-hour fast and a normal diet on the previous day. The samples were transported under refrigeration to the Nilson Lomanto Municipal Laboratory of Amargosa, Bahia, for analysis. Glucose levels were determined with an automatic biochemical analyzer (Biosystems^®^, model A15) by an enzymatic method based on the analysis of plasma fluoride. The presence of hyperglycemia was defined as a fasting glucose level ≥ 100 mg/dL as proposed by the American Diabetes Association (16).

### Statistical analysis

The data were analyzed using the SPSS 15.0 and MedCalc programs. Descriptive analysis consisted of the calculation of mean, median, standard deviation, percentile, and frequency. Differences in glycemic profile, BMI, WC and WHtR between sexes and age groups (children: 6 to 9 years; adolescents: 10 to 17 years) were tested by the Mann-Whitney test (p < 0.05). The partial correlation between the anthropometric indicators adjusted for sex and age was evaluated (p < 0.05). The prevalence ratio (PR), estimated by Poisson regression with robust variance, was used to verify the association of excess weight (overweight and obesity), elevated WC and high WHtR with hyperglycemia adjusted for sex, age and socioeconomic class. The Wald test was adopted to evaluate statistical significance (p < 0.05). The sensitivity, specificity and positive predictive value of excess weight (overweight and obesity) and high WC and WHtR were calculated. The power of the individual (BMI, WC, and WHtR) and combined indicators (BMI with WC and BMI with WHtR) to predict hyperglycemia was evaluated by constructing receiver operating characteristics (ROC) curves. The 95% confidence intervals (95% CI) were calculated and significance was attributed to areas under the ROC curve that showed a lower limit of the respective confidence intervals of 0.50 or higher. The difference in accuracy between the anthropometric indicators, alone or in combination, associated with hyperglycemia was calculated according to Hanley and McNeil (17).

## RESULTS

The number of students evaluated was 1,139, with 2.2% of losses due to refusal or absence on the day of data collection. The mean age was 11.51 years (3.33). Table 1 shows the differences in glycemia and anthropometric indicators according to sex and age group. Glycemia was higher in boys, and BMI, WC and WHtR were higher in girls. With respect to differences between children and adolescents, glycemia, BMI and WC were higher in adolescents, while WHtR was higher in children. The prevalence of hyperglycemia was 6.6% (95% CI 5.3-8.3). Young people with hyperglycemia had median (25^th^, 75^th^ percentiles) glycemia, BMI, WC and WHtR of 103 mg/dL (101, 106), 18.2 kg/m^2 ^(16.38, 20.28), 65.5 cm (60.0, 74.2) and 0.46 (0.42, 0.51), respectively. The prevalence of overweight was 12.7% (95% CI 10.9-14.8) and the prevalence of obesity was 3.2% (95% CI 2.3-4.3). High WC and WHtR were observed in 17.8% (95% CI 15.7-20.2) and 19.7% (95% CI 17.5-22.1) of the students, respectively.

**Table 1:** Glycemic profile and anthropometric indicators in the children and adolescents studied according to sex and age group. Northeastern Brazil, 2011-2012

	n	Glycemia (mg/dL)^a^	BMI (kg/m^2^)^a^	WC (cm)^a^	WHtR^a^
Sex					
Male	506	90 (85, 94)	17.00 (15.60, 19.40)	63.0 (56.5, 70.0)	0.44 (0.42, 0.46)
Female	633	88 (83, 93)	18.10 (15.90, 20.80)	66.9 (59.5, 73.6)	0.46 (0.43, 0.49)
p^b^		0.003	0.001	0.001	0.001
Age group					
Children (6 to 9 years)	363	88 (83, 93)	15.60 (14.60, 17.10)	56.4 (53.0, 60.4)	0.45 (0.43, 0.48)
Adolescents (10 to 17 years)	776	89 (85, 94)	18.80 (16.74, 21.20)	69.0 (63.4, 74.8)	0.45 (0.42, 0.48)
p^b^		0.033	0.001	0.001	0.041
Total	1.139	89 (84, 94)	17.50 (15.70, 20.10)	65.5 (58.0, 72.3)	0.45 (0.42, 0.48)

BMI: body mass index; WC: waist circumference; WHtR: waist-to-height ratio.

^a ^Median (25^th^, 75^th^ percentiles).

^b ^Significance level for glycemia, BMI, WC, and WHtR (Mann-Whitney test).

Strong correlations adjusted for age and sex were observed between all anthropometric indicators (BMI vs. WC = 0.87; BMI vs. WHtR = 0.87; WC vs. WHtR = 0.90). In multivariate logistic regression analysis adjusted for sex, age and socioeconomic condition, hyperglycemia was more likely to be present in young people with excess weight (PR = 1.70, 95% CI 1.03-2.85, p = 0.04), elevated WC (PR = 1.85, 95% CI 1.13-3.03, p = 0.01), and high WHtR (PR = 1.91, 95% CI 1.17-3.12, p = 0.01).

The sensitivity, specificity and positive predictive value were 24.3%, 84.5% and 10.0% for excess weight, 28.4%, 82.7% and 10.4% for WC, and 31.1%, 80.8% and 10.3% for WHtR, respectively. The accuracies to discriminate hyperglycemia were significant, but low, for all indicators studied. No significant differences were observed between individual and combined anthropometric indicators (Table 2).

**Table 2:** Accuracy of the anthropometric indicators for hyperglycemia screening in the children and adolescents studied. Northeastern Brazil, 2011-2012

Anthropometric indicator	AUC	95% CI
BMI (kg/m^2^)	0.56	0.53-0.59
WC (cm)	0.53	0.50-0.56
WHtR	0.55	0.52-0.58
BMI (kg/m^2^) and WC (cm)	0.55	0.52-0.58
BMI (kg/m^2^) and WHtR	0.55	0.52-0.58

BMI: body mass index; WC: waist circumference; WHtR: waist-to-height ratio; AUC: area under the curve; 95% CI: 95% confidence interval.

## DISCUSSION

The use of simple, easily obtainable and inexpensive measures such as body weight, height and WC for the screening of cardiometabolic risk factors in young people is a promising strategy for the prevention and early diagnosis of diseases such as type 2 diabetes. In this respect, the objective of this study was to evaluate the ability of BMI, WC and WHtR to discriminate hyperglycemia in the pediatric population, and to determine whether the inclusion of WC and/or WHtR increases the accuracy of BMI to discriminate hyperglycemia. Our results showed that the anthropometric indicators evaluated were associated with hyperglycemia. However, BMI, WC and WHtR were not efficient in screening for hyperglycemia in young people because their accuracy was poor. Furthermore, the addition of the indicators of abdominal obesity to BMI did not increase the ability of this index to discriminate young people with and without hyperglycemia.

In recent years, some authors have suggested the use of WC and WHtR for the evaluation of obesity and associated health problems in children and adolescents (6,7). However, others argue that there is not sufficient evidence to use these indicators instead of BMI (18,19). In fact, the average increase in the BMI of children and adolescents has been accompanied by an even more marked increase in WC (20). There might be additional advantages when this measure is corrected for height, such as the absence of a measurement unit, the lack of need for a specific reference population, and the possibility to use a single cut-off to discriminate excess fat in both young people and adults (WHtR = 0.5). However, evidence indicates that measuring height in addition to WC has no additional benefit for predicting cardiometabolic risk (21), and it may not be sufficient to adjust WC for height during the growth periods in childhood and adolescence because of the considerable residual correlation between height and WHtR (ranging from 0.29 to 0.36) (22). Furthermore, at least four different anatomical sites are commonly used to measure WC (23,24), a fact that can produce different prevalences of abdominal obesity (23) and impair the comparison between studies. Moreover, the magnitude of the association between WC and cardiometabolic risk factors in young people seems to be influenced by the site of measurement of this parameter (24).

With respect to the evaluation of general obesity, BMI has been the most used anthropometric indicator for decades. This index has wide applicability and is associated with less inter- and intraobserver error than demonstrated for other measures of obesity in young people (25). The calculation of BMI, which can be considered an obstacle to the use of this index by the population, can nowadays be performed easily using calculators available in electronic devices used in everyday life (*e.g*., mobile phones). Calculating BMI with a calculator can even be faster than to obtain other anthropometric measures of obesity. On the other hand, although children and adolescents with a high BMI also tend to have high levels of body fat, the BMI does not differentiate lean mass from fat mass and may therefore be considered an inaccurate indicator of body fat, especially among young people with normal or relatively low levels of body fat (26).

Previous studies analyzing anthropometric measures as predictors of hyperglycemia in children and adolescents also observed poor accuracies, ranging from 0.44 to 0.51 for BMI, from 0.44 to 0.52 for WC, and from 0.50 to 0.57 for WHtR (27-30). It is well known that excess body fat is associated with different physiological and pathological states, including alterations in glucose metabolism (31). In this respect, both the amount of total body fat and fat distribution exert moderate effects on glucose metabolism, with a greater effect being observed for abdominal visceral fat (31). Although attractive because they are noninvasive methods to estimate obesity, anthropometric indicators are not exact measures of total body fat and fat distribution (especially abdominal visceral fat) in children and adolescents (19). This limitation of the anthropometric method, in conjunction with the latency period of hyperglycemia, may explain at least in part the poor accuracies of BMI, WC and WHtR to discriminate hyperglycemia in young people. The phenomenon of “metabolically healthy obesity” observed among adults as well as children and adolescents (32) may also be a confounding factor in the relationship between anthropometric indicators and glycemia. Furthermore, factors not evaluated in the present study, such as the duration of obesity and the rate of recent weight gain/loss, may play an important role in altered glucose metabolism, irrespective of the current weight status (29).

Although the relationship between obesity and hyperglycemia/type 2 diabetes is well established in adults (33), in the pediatric population this subject requires further research. The PR obtained in this study indicated that overweight and obese young people were more likely to have hyperglycemia, irrespective of the anthropometric method used. Some studies also observed this association (7,34), while others did not demonstrate it (27,29). These divergences between the results of studies might be related to the characteristics of the samples such as age group, socioeconomic condition and lifestyle of the subjects studied, as well as methodological aspects, especially the criterion used to define hyperglycemia. In the present study, although a higher PR was observed for WHtR, the magnitude of the associations was similar (WHtR = 1.91, WC = 1.85 and BMI = 1.70). It should be mentioned that PR is not a statistical analysis that distinguishes between the presence and absence of disease; it only suggests an association between exposure and outcome and should therefore not be used to define whether a method is adequate to screen for a certain disease.

Strong correlations were observed in the present study between the three anthropometric indicators, in agreement with the findings of previous studies (18,19). Katzmarzyk and Bouchard (19) analyzed the correlation of total and visceral body fat with BMI and WC in children aged 5 to 18 years and found strong correlations between total and visceral body fat and the two anthropometric indicators. Accordingly, it can be expected that little additional information be obtained when adding WC and/or WHtR to BMI, as confirmed by our accuracy analysis. Chiolero and cols. (18) also observed that the addition of WHtR to BMI did not confer additional discriminatory power for elevated blood pressure in children. Taken together, the results suggest caution to state that WC or WHtR is a better substitute of BMI for the evaluation of body fat, or that the addition of these indicators to BMI improves the screening capacity for cardiometabolic risk in the pediatric population.

A strength of our study is the evaluation of the association of three simple anthropometric indicators with glycemia in a school-based probability sample consisting of children and adolescents of both genders from a developing country. Additionally, to our knowledge, there are no other studies investigating the addition of WC and WHtR to BMI in an attempt to improve the prediction of hyperglycemia in the pediatric population. However, this study has some limitation that need to be considered. A single measurement of fasting glucose levels, although suitable for populational studies, does not reflect the initial alterations in glucose homeostasis nor does it differentiate young people with and without impaired glucose tolerance/type 2 diabetes until significant deterioration in glucose metabolism has occurred (35). The cross-sectional design did not permit to establish cause-effect relationships since the exposure and outcome variables were collected simultaneously. Therefore, studies monitoring changes in body fat and glucose metabolism in young people over time are needed to gain further insight into this topic.

In conclusion, the anthropometric indicators studied were not useful to screen for hyperglycemia; however, obese children and adolescents were more likely to have hyperglycemia. In this respect and taking into consideration that obesity theoretically precedes hyperglycemia, BMI, WC and WHtR should continue to be used for the evaluation of overweight and obesity and consequent monitoring of metabolic diseases at early ages.
